# Multimodal In Vivo Imaging of Cutaneous Angiogenesis: A Structured Narrative Review of Dermoscopy, Dynamic OCT, and Line-Field Confocal OCT

**DOI:** 10.3390/diagnostics16213498

**Published:** 2026-10-26

**Authors:** Carmen Cantisani, Giovanni Paolino, Salvador Gonzalez, Gianluca Caruso, Ardeshir Bayat

**Affiliations:** 1 Complex Operative Unit (UOC) of Dermatology, Department of Clinical Internal and Cardiovascular Sciences, “Sapienza” University of Rome, 00161 Rome, Italy; 2 Unit of Dermatology, IRCCS Ospedale San Raffaele, Via Olgettina 60, 20132 Milan, Italy; paolino.giovanni@hsr.it; 3 Medicine and Medical Specialties Department, Alcala University, 28801 Madrid, Spain; salvagonrod@gmail.com; 4 Unit of Cellular Pathology, San Filippo Neri Hospital of Rome, 00135 Rome, Italy; gianluca.caruso@aslroma1.it; 5 Hair and Skin Research Laboratory, South African Medical Research Council (SAMRC) Wound Healing and Keloid Research Unit, Division of Dermatology, Department of Medicine, Faculty of Health Sciences, Groote Schuur Hospital, University of Cape Town, Cape Town 7700, South Africa

**Keywords:** angiogenesis, dermatology, line-field confocal optical coherence tomography, vascular phenotyping, optical imaging, structured narrative review, dermoscopy, dynamic optical coherence tomography, plexus depth

## Abstract

**Aims**: This structured narrative review aimed to synthesize and critically appraise the available human evidence on in vivo assessment of cutaneous angiogenesis using dermoscopy, dynamic optical coherence tomography (D-OCT), and line-field confocal optical coherence tomography (LC-OCT), and to determine whether disease-specific microvascular phenotypes can be defined across selected neoplastic and inflammatory dermatoses. Type of review. Structured narrative review (SNAR), using SANRA as a narrative-review quality framework. The work was not designed as a systematic review of diagnostic accuracy and was not prospectively registered. **Methods**: PubMed/MEDLINE, Scopus, and Web of Science were searched from inception to November 2025 using combinations of terms for angiogenesis, cutaneous vasculature, dermoscopy, OCT, D-OCT, LC-OCT, OCT angiography, melanoma, basal cell carcinoma, actinic keratosis, squamous neoplasia, Bowen disease, psoriasis, and Kaposi sarcoma. The clinical synthesis focused on original human studies; technical reviews and preclinical sources were used for background only. **Findings**: Findings were synthesized qualitatively because imaging protocols and vascular terminology were too heterogeneous for meta-analysis. Salient findings. Angiogenic architecture is disease-patterned rather than uniform. D-OCT studies associate melanoma vascular morphology with Breslow thickness, without establishing a validated optical staging test. Basal cell carcinoma shows hierarchically organized arborizing stromal vessels. Bowen disease shows glomerular, horizontally oriented units, whereas actinic keratosis shows milder, grade-dependent remodeling; multimodal imaging remains limited in separating hypertrophic actinic keratosis from early squamous cell carcinoma. Psoriasis shows elongated, regularly distributed dilated papillary loops, distinct from the less organized pattern of eczematous dermatitis. Kaposi sarcoma imaging evidence remains preliminary and does not establish stage-specific vascular criteria. Plexus depth is measurable and differs among some diseases and adjacent skin, but it is not a standalone marker of invasiveness. LC-OCT adds near-histologic three-dimensional morphology; D-OCT adds perfusion and depth; dermoscopy remains the accessible surface map. **Conclusions**: Multimodal in vivo imaging can link surface vascular clues to structural and functional substrates of cutaneous angiogenesis and can improve interpretive separation of disease-specific patterns from non-specific inflammatory remodeling. Most proposed quantitative biomarkers, including plexus depth, remain preliminary and require standardized descriptors and prospective multicenter validation, including studies with inflamed mimics, before routine clinical use.

## 1. Introduction

Angiogenesis is a fundamental biological process governing tissue homeostasis, regeneration, and immune surveillance, and its dysregulation constitutes a central mechanism in cancer progression, chronic inflammation, fibrotic remodeling, and wound repair. The skin, with its dense and highly specialized microvascular network, provides a unique and accessible model for in vivo investigation of angiogenic remodeling. However, comprehensive characterization of cutaneous angiogenesis remains technically challenging due to limitations in non-invasive imaging technologies, which restrict simultaneous assessment of microvascular morphology, spatial organization, and functional dynamics [1,2,3,4,5]. Pathological angiogenesis in dermatologic disease is characterized by reproducible alterations in microvascular architecture, including abnormal vessel caliber, tortuosity, hierarchical disorganization, shunting phenomena, and the formation of glomerular or polymorphic vascular structures [1,6,7,8,9]. These angiogenic phenotypes reflect disease-specific molecular drivers, such as vascular endothelial growth factor (VEGF) overexpression and endothelial activation, and are involved in the progression of cutaneous malignancies, chronic inflammatory diseases, impaired tissue repair, and photoinduced microvascular degeneration. A clinically useful distinction is whether angiogenesis is a primary pathogenic mechanism or a secondary reactive process.

Current in vivo diagnostic approaches, including dermoscopy, dynamic optical coherence tomography (D-OCT), and line-field confocal optical coherence tomography (LC-OCT), have contributed substantially to the study of skin angiogenesis. Dermoscopy has long served as the reference non-invasive technique for evaluating superficial vascular morphology; however, its two-dimensional perspective provides limited insight into the three-dimensional organization of cutaneous microvasculature and the underlying angiogenic mechanisms [10,11]. Recent advances in optical imaging have expanded the in vivo evaluation of skin vasculature, yet most techniques remain constrained by trade-offs between spatial resolution and penetration depth. As a result, critical angiogenic features remain insufficiently resolved in routine clinical and research settings. Line-field confocal optical coherence tomography (LC-OCT) represents a hybrid optical imaging technology designed to overcome these constraints by integrating confocal-level lateral resolution (~1 μm) with optical coherence tomography–based penetration (~500 μm), enabling near-isotropic, three-dimensional visualization of the cutaneous microvasculature in vivo [12,13,14]. Through rapid acquisition of orthogonal and volumetric datasets, LC-OCT permits detailed assessment of vascular lumen morphology, branching topology, and spatial distribution within the tissue.

In this review, we summarize and critically discuss the current evidence on the use of D-OCT and LC-OCT for the three-dimensional characterization of vascular architecture across a broad spectrum of dermatological conditions, comparing these findings with established dermoscopic vascular features [10,11]. By integrating available data on en face dermoscopic patterns with volumetric vascular imaging provided by D-OCT and LC-OCT, we highlight how these complementary modalities may improve the interpretation of disease-specific angiogenic mechanisms and identify imaging features with potential value as diagnostic, prognostic, and therapeutic biomarkers. Overall, this review aims to provide a comprehensive overview of the emerging role of three-dimensional vascular imaging as a complementary tool to dermoscopy, enhancing pathogenic understanding and supporting the development of angiogenesis-oriented, personalized therapeutic strategies.

Primary (driver) angiogenesis is exemplified by Kaposi sarcoma, in which endothelial proliferation itself constructs the lesion, and by tumor-associated neovascularization in invasive melanoma, where inefficient new vessels become part of progression. Secondary (reactive or supportive) angiogenesis accompanies epithelial expansion and inflammation: arborizing stromal vessels supporting basal cell carcinoma nests; glomerular remodeling beneath intraepidermal keratinocyte neoplasia; elongated papillary loops in psoriasis driven by the TNF–IL-23–IL-17 axis; and the more modest superficial vascular rarefaction or tortuosity of chronically ultraviolet-damaged skin. This distinction changes image interpretation. In primary angiogenic disease, disordered three-dimensional vascular geometry is the disease. In secondary angiogenesis, vessels are a readout of epithelial, inflammatory, or stromal activity and may normalize when the driving process is treated.

Dermoscopy, D-OCT, and LC-OCT. Dermoscopy magnifies the skin surface and the superficial vascular plexus and displays dotted, glomerular, arborizing, hairpin, linear irregular, and polymorphous vessels. Its strength is bedside pattern recognition; its constraint is an essentially two-dimensional en face view. D-OCT extracts motion contrast from moving erythrocytes and maps perfused vessels, with typical quantitative outputs of vessel density, plexus depth, diameter, and branching [15]. LC-OCT combines confocal-level lateral resolution (approximately 1 μm) with OCT-based penetration to about 500 μm, yielding near-isotropic three-dimensional datasets of lumen morphology, branching topology, and vessel–tissue relationships [12,14]. Reflectance confocal microscopy remains the reference for near-cellular horizontal imaging; it is complementary to, not interchangeable with, LC-OCT and D-OCT.

Previous studies. Dermoscopy has a mature evidence base for vascular clues in melanoma, basal cell carcinoma, Bowen disease, psoriasis, and Kaposi sarcoma [10,11]. D-OCT studies have quantified microvascular density and plexus depth in non-melanoma skin cancer [16], differentiated BCC subtypes by vascular architecture [17], characterized treatment-resistant actinic keratosis [18], and documented enlarged papillary loops in inflammatory disease [19]. LC-OCT series have described near-histologic correlates in BCC [20,21,22], actinic keratosis and squamous cell carcinoma [23], melanocytic lesions [24,25], psoriasis and other inflammatory dermatoses [26,27], and selected vascular lesions [28]. Three-dimensional segmentation of skin vasculature on LC-OCT is feasible [14]. What remained underdeveloped, and motivated this revision, is a cross-disease synthesis that links surface, functional, and volumetric findings into angiogenic phenotypes rather than isolated diagnostic signs.

Why D-OCT and LC-OCT add value, and at what cost. D-OCT and LC-OCT are not universally superior to dermoscopy. They are superior for questions that dermoscopy cannot answer, including plexus depth, three-dimensional branching, lumen irregularity, perfusion heterogeneity, and the relationship of vessels to tumor nests or elongated papillae. They are inferior for speed, cost, availability, field of view, and the immediately recognizable pattern language used in guidelines. Practical limitations include LC-OCT penetration limited to about 500 μm, operator dependence, degradation by hyperkeratosis, crust, ulceration, motion, and marked pigmentation, small fields of view, and non-standardized vascular vocabularies. These constraints explain why the techniques remain adjunctive specialist tools rather than replacements for dermoscopy or histopathology.

Limitations of existing modalities and the biomarker proposition. Dermoscopy recognizes surface patterns without reliable microstructural correlation. Many OCT-angiography implementations provide flow without near-cellular structure. Histology remains the gold standard for vessel–tissue relationships but is destructive and poorly suited to serial monitoring. The proposition that D-OCT and LC-OCT may yield diagnostic, prognostic, and therapeutic biomarkers is biologically plausible but only partly evidenced. Candidate metrics include vessel density, diameter distribution, branching complexity, tortuosity, caliber heterogeneity, plexus depth, lumen continuity, and perfusion asymmetry. Throughout this revision we distinguish descriptors that are repeatedly observed from biomarkers that remain hypothetical.

Novel contribution and rationale for this structured narrative review. The novel contribution of this review is not a new device, but a disease-spanning angiogenic reading of already available multimodal images: (i) dermoscopy, D-OCT, and LC-OCT as complementary layers of one vascular phenotype; (ii) primary versus secondary angiogenesis; (iii) stage or subtype comparisons (melanoma thickness-associated observations; uncertainties in Kaposi sarcoma staging; actinic keratosis morphology; psoriasis versus eczematous dermatitis); and (iv) explicit grading of biomarkers as established, emerging, or hypothetical. A structured narrative rather than a systematic diagnostic accuracy review was chosen because source studies are heterogeneous in design, terminology, and outcomes, formal pooling would be misleading, and the clinical need is a critical conceptual synthesis. The rationale is translational: if angiogenesis can be visualized repeatedly and non-invasively, dermatology can move from static surface signs toward monitoring of a dynamic, potentially targetable biological process.

## 2. Methods

This structured narrative review examines human evidence on dermoscopy, dynamic optical coherence tomography (D-OCT), and line-field confocal optical coherence tomography (LC-OCT) for cutaneous vascular imaging. The manuscript reports searches of PubMed/MEDLINE, Scopus, and Web of Science from inception to November 2025, supplemented by reference-list screening. Search concepts covered angiogenesis, skin vasculature, the imaging modalities, and the selected neoplastic, inflammatory, and vascular disorders. The review was not prospectively registered. Findings were synthesized qualitatively because study designs, imaging protocols, and vascular terminology were heterogeneous. SANRA was used as a narrative-review quality framework; this does not constitute a systematic review or demonstrate compliance with PRISMA-ScR.

The clinical synthesis focuses on original human cutaneous imaging studies, including observational studies, pilot studies, case series, and case reports reporting vascular findings. Technical reviews, foundational angiogenesis literature, and preclinical studies are cited for background only and are not treated as primary human clinical evidence. Sources without relevant cutaneous imaging findings, duplicate reports, editorials, and conference abstracts without adequate methodological detail were excluded from the clinical synthesis. The manuscript describes English-language selection and extraction of study design, condition, modality, sample size, vascular observations, and clinical implications. No pooled diagnostic estimates or formal scored risk-of-bias assessment were produced. Disease selection provides examples of melanocytic and keratinocyte neoplasia, inflammatory papillary remodeling, and endothelial neoplasia. Differences in evidence strength and the exploratory nature of proposed biomarkers are considered in the disease-specific discussion.

## 3. Results

Findings are presented below by disease, with original multimodal figures from the authors’ clinical archives of consenting patients examined during routine care using commercially available dermoscopy, D-OCT, and LC-OCT systems. These images are illustrative correlates of the literature synthesis, not a new prospective cohort, and were not taken from previously copyrighted publications.

### 3.1. Melanoma

**Figure 1:**
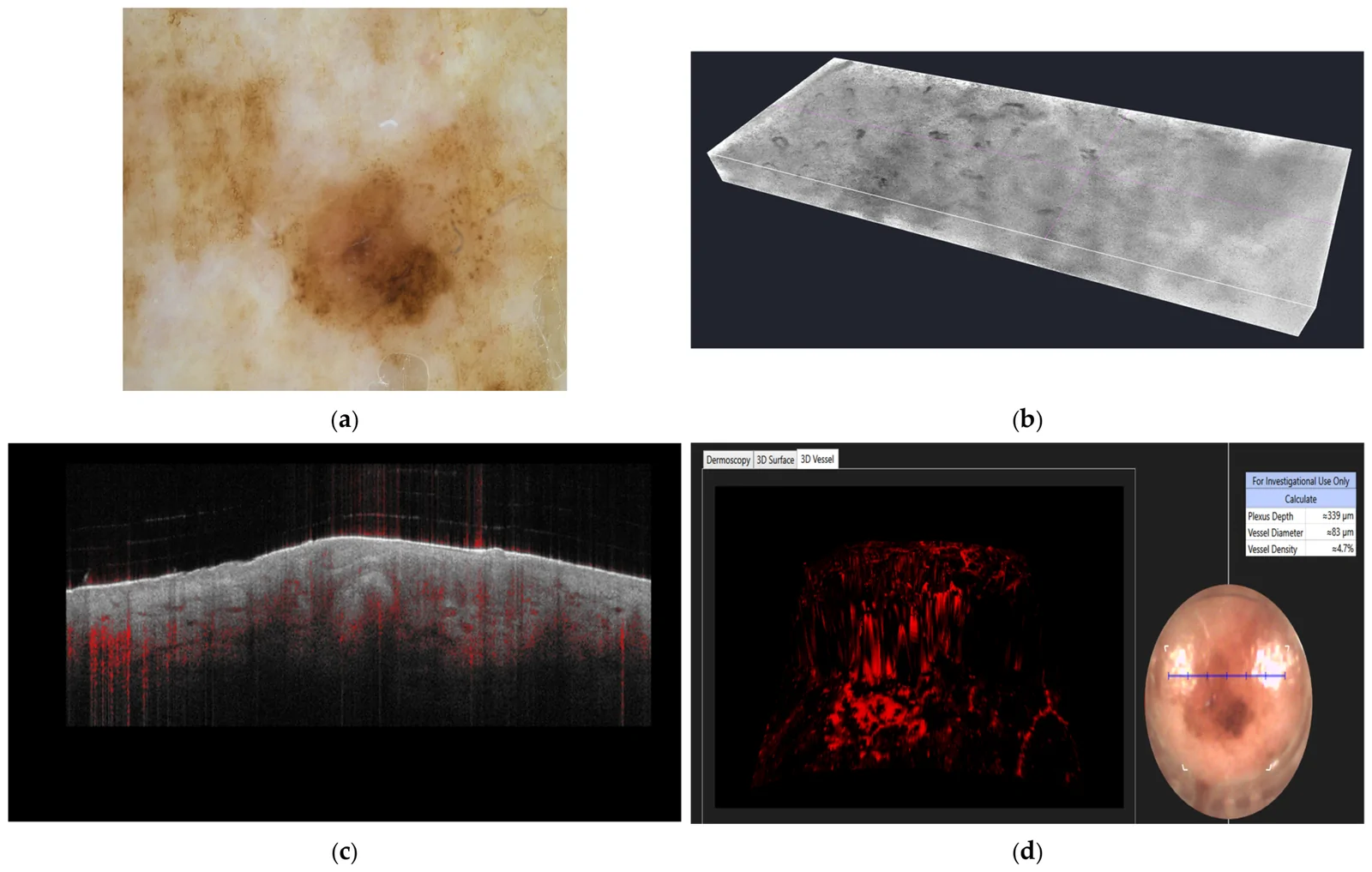
Illustrative multimodal appearances of melanoma. (**a**) Dermoscopy. (**b**) Structural LC-OCT imaging. (**c**,**d**) OCT and vascular imaging views. Panel assignments, acquisition modes, and any displayed measurements require verification against the original images. Structural vessel appearances alone do not establish flow velocity, hypoxia, or invasion.

Dermoscopy may reveal atypical linear, dotted, or polymorphous vessels in melanoma [10]. Human D-OCT studies report associations between vascular morphology and Breslow thickness [29]. These associations do not establish a non-invasive staging test. LC-OCT studies of melanocytic lesions primarily describe cellular and architectural criteria [24,25]; structural vascular appearances should not be interpreted as direct measurements of perfusion, tissue hypoxia, or tumor invasion. Figure 1 is intended to illustrate complementary imaging appearances, subject to confirmation of the image panels and their provenance.

### 3.2. Basal Cell Carcinoma (BCC)

**Figure 2:**
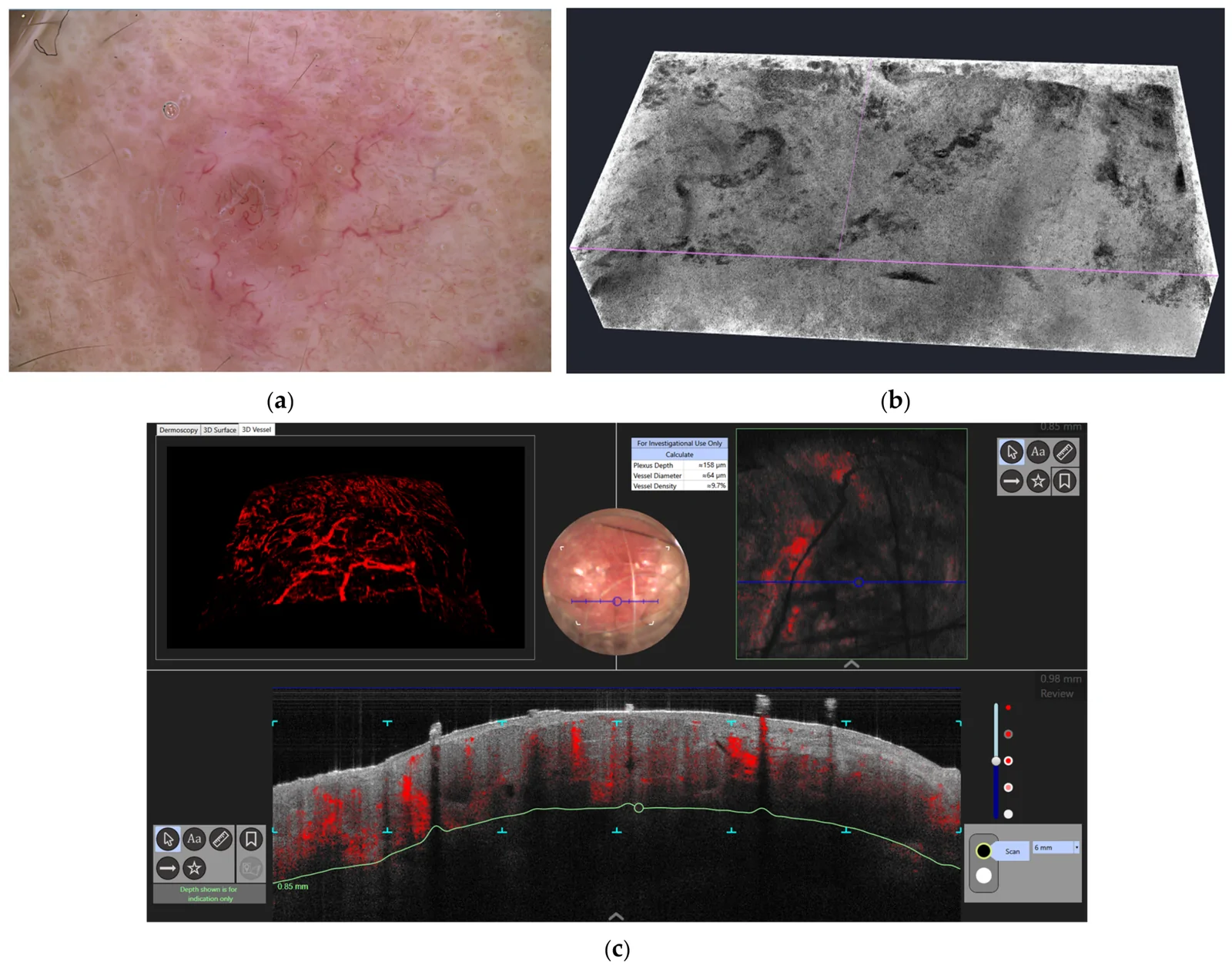
Illustrative multimodal appearances of basal cell carcinoma. (**a**) Dermoscopy. (**b**) LC-OCT structural assessment of tumor lobules and adjacent vascular spaces. (**c**) OCT vascular imaging. Panel assignments require confirmation against the original images; structural dark spaces must not be equated with perfused vessels without appropriate flow-sensitive imaging.

In BCC, LC-OCT identified arborizing vessels with hierarchical branching penetrating the tumor stroma, frequently surrounding hyporeflective tumor islands [20,21,22]. Three-dimensional visualization may reveal structural vascular details not apparent dermoscopically, including micro-branching within primary arborizing vessels and variation in vessel caliber. These volumetric features align with VEGF-mediated but structurally organized angiogenesis, offering potential quantitative markers for subtype differentiation and monitoring of therapeutic response, such as vascular regression following topical or systemic interventions [17,21]. BCC shows characteristic tumor conglomerates with a signal intensity similar to that of the epidermis [15,17]. Superficial tumor nests derive from the epidermis, while deeper parts of the BCC are sharply demarcated from the dermis by a dark line, correlating with the surrounding fibrous stroma (Figure 2). Typically, the tumor is accompanied by enlarged blood vessels. Supporting studies include D-OCT subtype mapping and LC-OCT–histopathology and diagnostic-confidence series [17,20,21,22]. Use of branching metrics for robust subtype separation and treatment monitoring remains promising but not fully validated.

### 3.3. Actinic Keratosis

Actinic keratosis and early squamous neoplasia represent another clinically relevant setting in which vascular imaging may prove particularly informative. In these lesions, the coexistence of epidermal atypia, parakeratosis, and superficial vascular remodeling reflects a transition from chronic photodamage to keratinocytic neoplasia [18,23,30]. D-OCT has shown potential in identifying vascular patterns associated with treatment-resistant or higher-risk lesions, while LC-OCT may provide more refined structural correlation by visualizing epidermal disarray, disruption of the dermo–epidermal junction, and the arrangement of glomerular or irregular vascular units within the superficial dermis [16,18,23,30]. This is especially relevant in the context of field cancerization, where the distinction between actinic keratosis, Bowen disease, and early invasive squamous cell carcinoma may be clinically subtle yet biologically meaningful. From this perspective, multimodal vascular imaging could contribute not only to lesion-level assessment, but also to the non-invasive characterization of chronically UV-damaged skin as a pro-angiogenic and pro-neoplastic microenvironment [9,31,32]. Direct D-OCT/LC-OCT comparisons remain sparse. Atrophic lesions tend to show a thinner epidermis, more readily visualized superficial dotted or short linear vessels, and a relatively preserved although often flattened dermo–epidermal junction. Hypertrophic lesions show compact hyperkeratosis and acanthosis that may attenuate optical penetration; vessels appear more irregular or clustered and are more often accompanied by marked epidermal disarray. Current diagnostic limitation is clear: multimodal imaging cannot reliably replace biopsy when the question is early invasion. Interrupted dermo–epidermal junction and bizarre nodular vascular clusters raise concern for squamous cell carcinoma, but hyperkeratosis, inflammation, and overlapping glomerular patterns produce both false reassurance and false alarm. Distinguishing actinic keratosis, Bowen disease, and early invasive squamous cell carcinoma therefore remains a limitation of multimodal imaging rather than an established strength.

### 3.4. Bowen Disease

**Figure 3:**
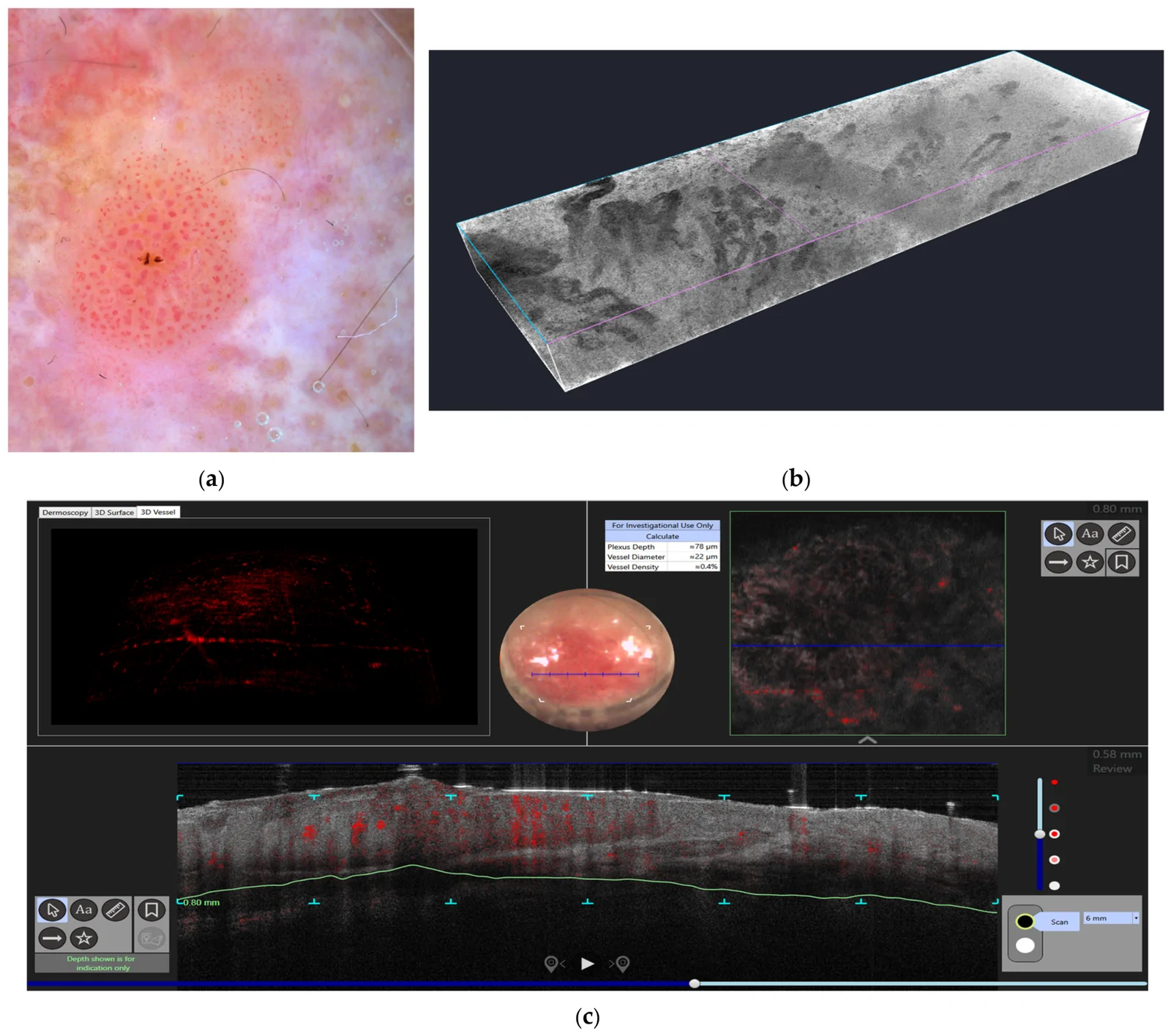
Illustrative multimodal appearances of Bowen disease. (**a**) Dermoscopy showing glomerular vessels. (**b**) LC-OCT structural imaging. (**c**) OCT vascular imaging. The images and panel assignments require confirmation; junctional non-visualization must be distinguished from proven invasion.

LC-OCT assessment of Bowen disease has described epidermal keratinocyte atypia with dilated, horizontally oriented microvessels and glomerular vessels (Figure 3). The three-dimensional perspective revealed deeper vascular elongation into the upper dermis, which is not appreciable via dermoscopy. Vessel density and tortuosity metrics derived from volumetric imaging provide potential indicators of early neoplastic angiogenic activation, useful for monitoring lesion progression or response to topical treatments [23,30,33]. The proposition that vessel density and tortuosity are biomarkers for Bowen disease is preliminary. It is biologically attractive and consistent with three-dimensional morphology, but it has not been locked to progression or treatment-outcome endpoints in prospective multicenter studies and is treated here as a future-direction candidate rather than a clinical biomarker. Bowen disease is squamous cell carcinoma in situ. An indistinct or non-visualized dermo–epidermal junction on optical imaging does not establish invasion; suspected invasion requires histopathological assessment.

### 3.5. Psoriasis

**Figure 4:**
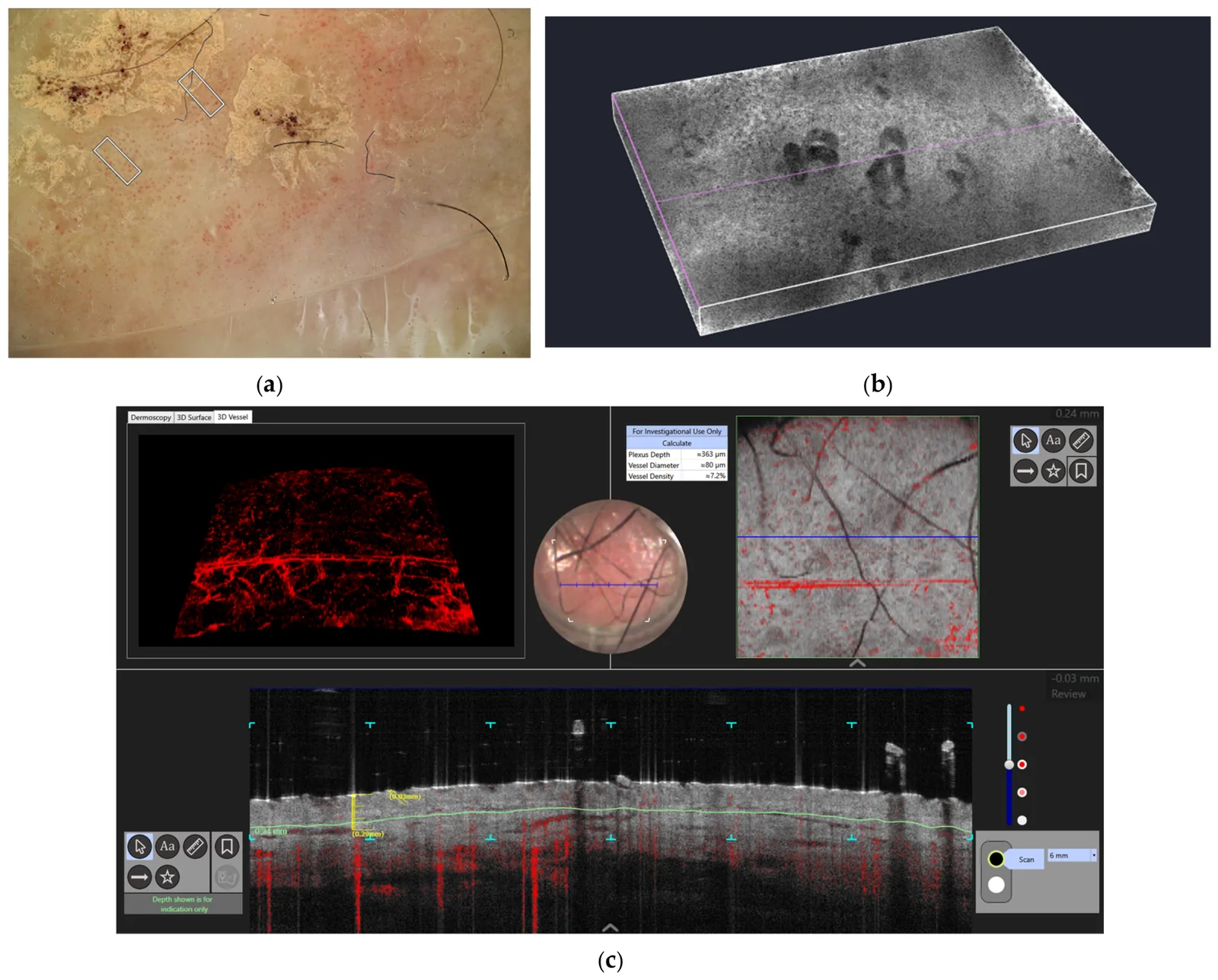
(**a**) Dermoscopic appearance with an inflammatory glomerular vessel pattern. (**b**) LC-OCT: Homogeneous vessels of similar length and papillae of similar thickness. Vessels are spread regularly around the surface of the lesion. (**c**) D-OCT: Increase in vessel density and subtle branching bulging symmetric pattern, without atypical keratinocytes or neoplastic hypertrophic skin, inflammatory papillomatous epidermal appearance.

LC-OCT descriptions of psoriasis show elongated dermal papillae containing dilated capillary loops, with histopathological correlation in a small inflammatory-disease case series [26]. The cited evidence does not establish a reproducible percentage increase in LC-OCT vessel density. A separate OCT report followed four patients with psoriasis or atopic dermatitis during biologic treatment and explored epidermal and vascular parameters over 52 weeks [19]. Those observations should not be transferred to LC-OCT as validated functional biomarkers. Psoriasis may show more regular papillary elongation and capillary loops than eczematous dermatitis, but mixed or treated phenotypes overlap. These features remain supportive adjuncts rather than standalone diagnostic or treatment-response criteria (Figure 4).

### 3.6. Kaposi Sarcoma

**Figure 5:**
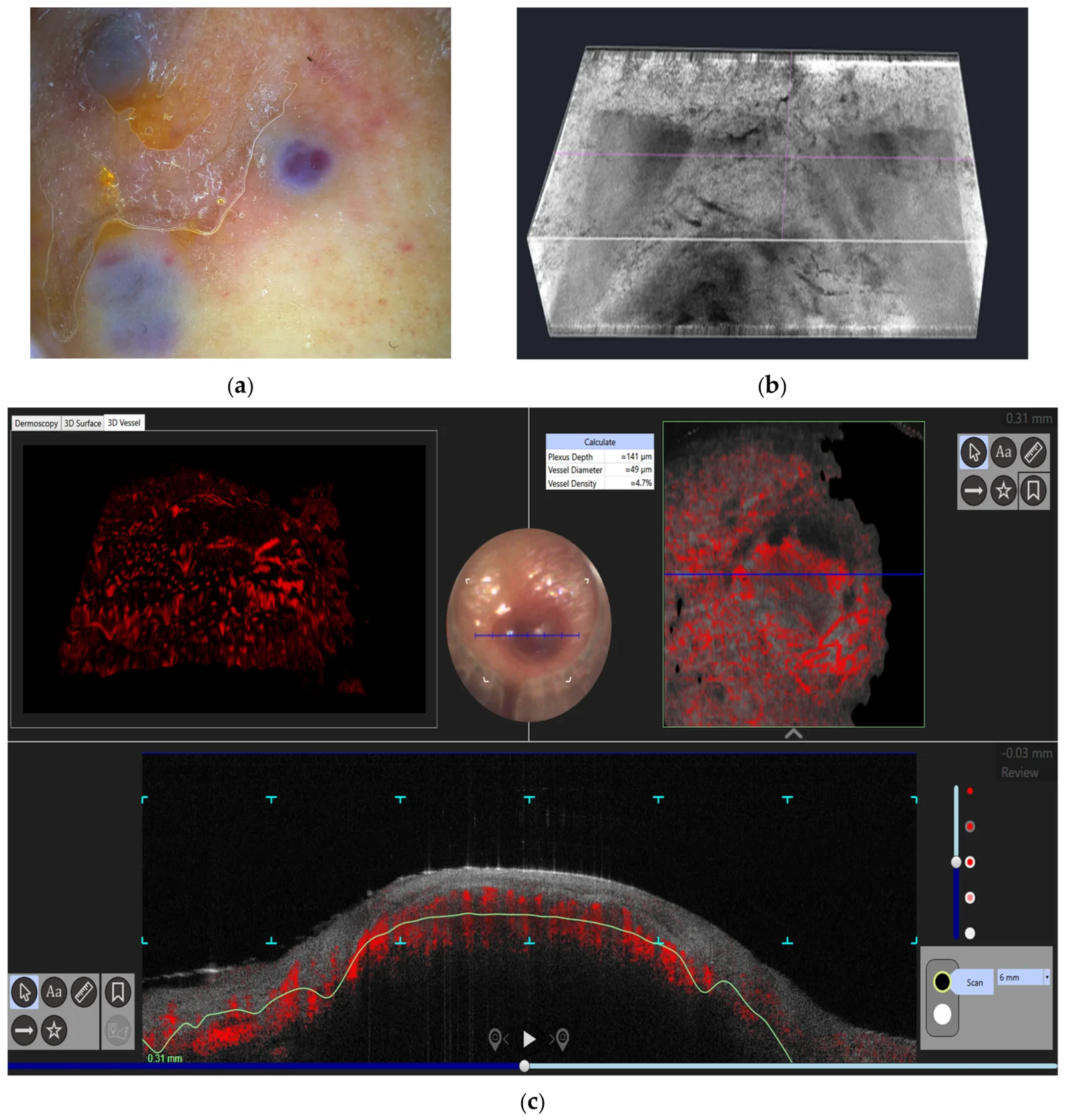
Illustrative multimodal appearances of Kaposi sarcoma. (**a**) Dermoscopy. (**b**) LC-OCT structural imaging. (**c**) OCT vascular imaging. Confirm the original panels and acquisition modes. Optical appearances should not be described as proof of atypical endothelial spindle cells without histopathological correlation.

Published LC-OCT descriptions of vascular lesions and a Kaposi sarcoma imaging report provide preliminary morphological observations [28,34]. These sources do not establish validated OCT criteria for distinguishing patch, plaque, and nodular stages or selecting treatment. Structural channels and complex vascular appearances may be useful research descriptors (Figure 5), but cannot alone establish endothelial proliferation, disease activity, or therapeutic response. Stage-specific performance requires comparative studies with histopathological correlation, reproducibility assessment, and clinical outcome validation.

### 3.7. Key Observations Across Cases

1. LC-OCT can depict small structural vascular spaces and vessel–tissue relationships; identification of endothelial buds or microaneurysms requires appropriate correlation and should not be assumed from resolution alone [12,14,28].
2. Volumetric imaging allowed differentiation of organized vs. chaotic angiogenesis, correlating with disease behavior and therapeutic response potential (Table 1).
3. Motion-contrast-derived perfusion maps highlighted functional heterogeneity, offering prospective biomarkers for early intervention and precision therapy.
4. Three-dimensional vascular phenotyping enhanced interpretability of superficial dermoscopic patterns, bridging morphological assessment with pathophysiological insight.

Plexus depth is the distance from the skin surface, or from a fitted epidermal contour, to the first reliably detected perfused vessels. D-OCT customarily samples this anatomy at 150, 300, and 500 μm. Published D-OCT series of thicker melanomas show dotted loops still dominant at 150 μm, whereas blobs, coils, and serpiginous vessels become significantly more frequent at 300 and 500 μm as Breslow thickness increases [29]. In the keratinocyte-cancer spectrum, vessels are on average more superficial than in matched healthy skin. Sigsgaard et al. [16] measured surface-to-first-vessel distance in 52 histologically verified lesions (13 actinic keratoses, 22 BCCs, 17 SCCs) and found a significant difference between non-melanoma skin cancer and adjacent normal skin, a significant difference for BCC versus control, and only a borderline difference between actinic keratosis and SCC. Themstrup et al. [30], scoring blinded en face D-OCT frames at 150, 300, and 500 μm, showed that morphology at a stated depth is more discriminative than depth alone: strong “blob” vessels at 300 μm favored Bowen disease, whereas curved vessels were more typical of actinic keratosis. In inflammatory disease the relevant depth signal is often loop elongation rather than downward migration of the entire plexus. Psoriasis contains vertically stretched dilated capillaries; treatment studies have reported change in plexus depth together with density and diameter [19]. Eczematous dermatitis tends to show milder, less regularly elongated loops. Stage-specific depth differences in Kaposi sarcoma remain hypotheses requiring comparative validation. Plexus depth is a real, device-measurable quantity and a plausible companion marker. It is not yet a standalone scale of invasiveness. A shorter surface-to-first-vessel distance can mean true upward angiogenesis, epidermal thinning, or optical artifact from scale. A longer distance can mean invasion into deeper dermis or simple acanthosis. Depth must be interpreted with vessel shape at standardized planes, epidermal thickness, body site, and adjacent normal skin.

Disease-specific angiogenic patterns versus non-specific inflammatory remodeling. Disease-specific patterns are recognized when three layers agree: a dermoscopic silhouette, a D-OCT depth-and-flow map, and an LC-OCT three-dimensional relationship between vessels and epithelium or stroma. Specific signatures in this review are hierarchical arborizing trunks that wrap hyporeflective BCC islands; chaotic serpiginous channels with caliber jumps and avascular lacunae in invasive melanoma; glomerular horizontal coils under atypical Bowen epidermis; spiraliform overlapping channels and microaneurysms in Kaposi sarcoma; and regular dilated papillary loops in psoriasis. Vascular patterns overlap with reactive and inflammatory conditions and are not individually specific for neoplasia. Non-specific inflammatory or remodeling change tends to be reactive: vessels enlarge where epidermis is acanthotic or dermis is edematous, but they keep a recognizable hierarchy (loops in papillae, a still-traceable subpapillary plexus). Inflamed seborrheic keratoses, traumatized nevi, granulation tissue, and ulcerated tumors can still produce dotted or polymorphous surface vessels. Shared VEGF-driven dilatation occurs in both psoriatic papillae and tumor stroma. Multimodal imaging therefore improves interpretive specificity relative to dermoscopy or redness alone; it does not abolish overlap and cannot, on present evidence, certify that a vascular change is neoplastic rather than inflammatory without the structural and clinical context of the lesion.

## 4. Discussion

This review highlights the growing relevance of non-invasive vascular imaging in dermatology and supports the concept that cutaneous angiogenesis can be interrogated in vivo with increasing structural and functional detail by integrating dermoscopy, dynamic optical coherence tomography (D-OCT), and line-field confocal optical coherence tomography (LC-OCT) [12,13,15]. Across inflammatory, neoplastic, and vascular skin disorders, the available evidence suggests that angiogenesis is not merely an accompanying epiphenomenon, but a biologically meaningful component of disease architecture that can now be explored beyond the limits of conventional surface inspection. In this context, LC-OCT appears particularly promising because it bridges the gap between superficial vascular pattern recognition and near-histologic three-dimensional assessment of the cutaneous microvasculature. By combining high lateral resolution with volumetric imaging, LC-OCT makes it possible to visualize not only the presence of vessels, but also their caliber, branching topology, lumen continuity, spatial orientation, and relationship with the surrounding epidermal and dermal structures. This represents a substantial conceptual advance in the study of skin angiogenesis, because it allows vascular morphology to be interpreted as a dynamic component of disease biology rather than as a purely descriptive sign. Melanoma progresses from sparse junctional dotted flow to chaotic caliber-variable networks; BCC maintains hierarchical arborizing stroma; actinic keratosis shows grade- and thickness-dependent remodeling that does not yet reliably separate invasion; Bowen disease converts the glomerular dermoscopic dot into a deeper-reaching microvascular unit; psoriasis displays regular dilated papillary loops that differ from eczematous dermatitis; Kaposi sarcoma has preliminary structural imaging descriptions, with stage-specific vascular criteria still unvalidated.

A major message emerging from the present review is that dermoscopy, D-OCT, LC-OCT, and reflectance confocal microscopy (RCM) should not be considered competing modalities, but rather complementary tools that interrogate different layers of the angiogenic process. Dermoscopy remains the cornerstone of vascular evaluation in routine dermatology because it is rapid, inexpensive, widely available, and highly effective in identifying superficial vascular patterns such as dotted, glomerular, arborizing, hairpin, linear irregular, or polymorphous vessels. In many clinical settings, these patterns already provide substantial diagnostic value and often represent the first clue to the biologic nature of a lesion. However, dermoscopy is inherently limited by its en face and two-dimensional perspective, which does not allow reliable assessment of vascular depth, three-dimensional branching, vessel–stroma relationships, or the architecture of deeper dermal vascular plexuses. Consequently, dermoscopy can suggest the presence of angiogenic remodeling, but cannot fully resolve its structural organization. This limitation becomes especially relevant in lesions where the same superficial vascular clue may correspond to very different underlying microvascular arrangements, or where critical angiogenic changes occur beneath an only subtly altered dermoscopic surface.

D-OCT expands this framework by introducing a functional dimension to cutaneous vascular imaging. Through motion-contrast analysis of moving erythrocytes, D-OCT enables in vivo visualization of blood flow and allows assessment of vessel density, plexus depth, branching arrangement, and perfusion-related vascular maps within the superficial dermis. This is a major step beyond dermoscopy, because it transforms the evaluation of vessels from a purely morphologic exercise into a partially hemodynamic one. In inflammatory diseases such as psoriasis, small OCT studies have explored papillary vessels and vascular parameters during treatment; these remain exploratory observations [15,19]. In epithelial neoplasms and keratinocyte carcinomas, D-OCT may help characterize vascular support patterns and may provide clues regarding lesion aggressiveness or treatment-induced vascular regression. Nevertheless, D-OCT still involves a trade-off between penetration and spatial resolution. While it can depict perfused vascular networks and provide valuable information on their depth and distribution, it does not consistently resolve the finest morphologic details of microvascular remodeling, such as very small endothelial buds, subtle lumen irregularities, microaneurysmal changes, or the precise relationship between vessels and tumor nests or dysplastic epidermal structures. It is precisely at this interface that LC-OCT adds value.

LC-OCT provides near-cellular structural detail and volumetric views of superficial skin [12,13,14]. Dark vascular spaces on structural LC-OCT do not by themselves demonstrate perfusion. D-OCT supplies complementary motion-contrast information about perfused vessels. Evidence of improved diagnostic confidence for equivocal BCC [22] concerns lesion diagnosis and does not establish superiority for vascular biomarkers. Comparison with RCM in keratinocyte tumors likewise does not validate a general hierarchy of vascular imaging performance [32]. The appropriate modality depends on the clinical question, depth, resolution, and need for functional or structural information.

Reflectance confocal microscopy occupies another important position in this imaging spectrum and deserves explicit comparison. RCM has long been one of the most powerful non-invasive tools for near-histologic assessment of the epidermis and superficial dermis, particularly in melanocytic lesions, basal cell carcinoma, and inflammatory dermatoses [13,32]. It provides exquisite horizontal optical sections and allows detailed visualization of cellular morphology, epidermal architecture, pagetoid spread, inflammatory infiltrates, and, to some extent, superficial vascular structures. In the context of angiogenesis, RCM can reveal dilated horizontal vessels, increased vascular density in inflammatory disorders, leukocyte trafficking, and vascular-associated changes in the papillary dermis. However, the vascular assessment provided by RCM remains partial. The technique is exquisitely sensitive for superficial horizontal imaging, but is more limited in penetration depth and in the ability to reconstruct continuous three-dimensional vascular trajectories across the dermo–epidermal junction and upper dermis. In other words, RCM is extremely strong in the cytologic and microarchitectural characterization of the tissue environment in which angiogenesis occurs, but less suited than LC-OCT or D-OCT to mapping the full three-dimensional organization of vascular networks over depth. For this reason, RCM and LC-OCT should be seen as highly complementary: the former excels in cellular and epidermal detail, whereas the latter adds volumetric and vertical structural context, especially for the vascular compartment.

The disease-specific findings summarized in this review further support the notion that angiogenesis in dermatology is not a single stereotyped process, but a spectrum of biologically distinct remodeling programs. Melanoma provides perhaps the clearest example of disordered tumor-associated angiogenesis. Across the available literature, melanocytic lesions with malignant potential tend to show irregular vascular distribution, polymorphous superficial patterns, and increasing microvascular complexity with progression. Dermoscopy often captures this as atypical linear vessels, milky-red areas, dotted vessels, or polymorphous vascular patterns, but such surface clues are only the visible endpoint of a deeper and more heterogeneous process. D-OCT studies suggest that melanoma may exhibit altered vascular density, irregular distribution, and changes in plexus depth [29], while LC-OCT adds the possibility of visualizing abrupt caliber changes, serpiginous vessel trajectories, focal crowding, and avascular gaps within the same lesion. These features are biologically plausible because melanoma angiogenesis is typically driven by hypoxia, VEGF signaling, inflammatory stromal crosstalk, and spatially heterogeneous tumor growth. The coexistence of crowded vessels and relatively hypoperfused areas may reflect inefficient tumor angiogenesis, in which newly formed vessels are structurally abnormal and unevenly distributed. From a translational standpoint, this is important because vascular heterogeneity itself may become an in vivo biomarker of tumor aggressiveness, invasion, or microenvironmental instability. In this setting, RCM can contribute by identifying atypical junctional architecture, pagetoid spread, and superficial vascular clues, but LC-OCT and D-OCT appear better suited to explore how vascular remodeling extends below the dermoscopic surface and interacts with invasive growth. Areas of disagreement and limited evidence include the following: devices differ, pigmentation and regression confound flow contrast, and “polymorphous vessels” on dermoscopy can correspond to more than one volumetric geometry. Claims that LC-OCT vascular crowding is a functional biomarker of melanoma aggressiveness remain provisional.

Basal cell carcinoma illustrates a different angiogenic phenotype, one that is more structured and driven by tumor–stroma interaction. Arborizing vessels are among the best-established dermoscopic hallmarks of BCC and often already suggest the diagnosis with high confidence. Yet the biologic value of volumetric imaging lies in showing that these vessels are not merely superficial tree-like structures, but components of a fibrovascular support network surrounding and penetrating tumor nests. D-OCT can reveal enlarged vessels and flow-associated branching patterns, while LC-OCT can depict the relationship between vascular structures and hyporeflective tumor islands, the hierarchical branching of arborizing vessels, and subtle differences in vascular arrangement that may correlate with superficial, nodular, or infiltrative subtypes. This is clinically relevant because BCC angiogenesis is generally less chaotic than that of melanoma and more closely linked to a relatively organized tumor–stroma interaction. Such organization may explain why BCC often displays highly recognizable dermoscopic vascular morphology. At the same time, it suggests that LC-OCT-derived vascular descriptors (such as branching complexity, vessel diameter distribution, or vessel–tumor nest proximity) might eventually assist in subtype stratification or in monitoring vascular regression during topical, photodynamic, or systemic treatment. RCM, by contrast, remains particularly valuable for identifying classic BCC criteria such as elongated tumor islands, peripheral palisading, clefting, and horizontal vessels in the superficial dermis, but again provides less direct three-dimensional vascular mapping than LC-OCT. Disagreement appears when branching complexity is converted into a subtype classifier: infiltrative BCC may have finer, less obviously arborizing vessels, and inflammation can mimic dilatation. Vascular regression during topical or photodynamic therapy is documented in small series and is a research endpoint, not yet a decision rule.

Bowen disease and actinic keratosis occupy an especially interesting position because they lie at the interface between inflammatory photodamage, intraepidermal neoplasia, and early angiogenic activation. Dermoscopically, glomerular vessels are among the best-known vascular clues of Bowen disease, whereas actinic keratosis may display a variable combination of erythematous pseudo-network, rosettes, targetoid follicles, and superficial vascular changes depending on grade and background photodamage. These findings are clinically useful, but they do not fully explain how the vascular compartment remodels during the transition from dysplasia to overt intraepidermal carcinoma and, eventually, invasive squamous neoplasia. D-OCT can contribute by demonstrating increased vascularity and altered superficial perfusion patterns, while LC-OCT appears capable of linking these changes to epidermal atypia, assessment of the dermo–epidermal junction, and horizontally oriented or glomerular microvascular structures extending into the upper dermis. In this sense, LC-OCT may help reinterpret the classic dermoscopic “glomerular vessel” as the surface correlate of a more complex three-dimensional microvascular configuration associated with intraepidermal neoplastic proliferation. This could be particularly valuable in field cancerization, where chronically UV-damaged skin contains a mosaic of subclinical vascular and keratinocytic abnormalities that are difficult to stage using surface morphology alone. RCM is also useful in this setting because it can reveal keratinocyte atypia, architectural disarray, and inflammatory changes in sun-damaged skin, but again its strength lies more in cytologic and superficial microarchitectural characterization than in full-depth vascular mapping. This is the widest gap between biological plausibility and diagnostic performance. Variability in the literature reflects different devices, different definitions of “glomerular,” and selected rather than consecutive lesions. Until prospective studies with predefined criteria and histopathologic blinding are available, multimodal imaging should refine, not replace, biopsy decisions on the squamous spectrum.

Psoriasis, by contrast, represents the paradigm of inflammatory angiogenesis rather than tumor-associated neovascularization. The elongated, tortuous, and dilated capillary loops within psoriatic dermal papillae are among the best-known microvascular alterations in inflammatory skin disease and are tightly linked to keratinocyte hyperproliferation, endothelial activation, and the cytokine axis involving TNF, IL-17, and IL-23. 

Dermoscopy often visualizes these changes as regularly distributed dotted vessels over a light red background, a pattern that is highly useful in clinical diagnosis. Yet dermoscopy cannot quantify how those dotted vessels relate to the papillary vascular unit in three dimensions, nor can it adequately capture subtle differences in perfusion or branching asymmetry. This is where D-OCT and LC-OCT become particularly attractive. D-OCT can visualize enlarged papillary loops, increased vessel density, and perfusion changes, while LC-OCT may further resolve the three-dimensional arrangement of dilated capillary loops within elongated papillae and detect more subtle irregularities in branching or vessel morphology. Such information is not merely descriptive: vascular normalization is increasingly recognized as an important component of therapeutic response in psoriasis, and imaging-derived vascular metrics may therefore serve as early biomarkers of biologic efficacy. RCM also has a strong role in psoriasis, particularly for visualizing papillomatosis, parakeratosis, inflammatory cell trafficking, and dilated capillaries in the papillary dermis. However, when the specific question concerns the three-dimensional vascular architecture of inflammatory papillae and its evolution over time, LC-OCT and D-OCT may offer a more direct view of the angiogenic component of disease. Head-to-head sample sizes versus eczema are small. Variability also arises from body site, acanthosis thickness, and whether authors score loops, density, or perfusion. Vascular metrics are best framed as activity companions to clinical scores, not as replacements for them.

Kaposi sarcoma is an endothelial neoplasm for which multimodal imaging may provide complementary structural observations. Published evidence remains preliminary [34]. Optical vascular morphology should not be presented as a validated measure of histological stage, spindle-cell proliferation, disease activity, or response to therapy. Comparative studies with histopathological correlation and predefined stage criteria are required before such applications can inform treatment.

Taken together, these observations suggest that one of the most important future applications of LC-OCT and D-OCT lies in the development of biomarker-oriented vascular phenotyping. The goal is not simply to produce increasingly sophisticated images of vessels, but to extract reproducible, clinically meaningful descriptors of angiogenesis. Candidate metrics include vessel density, lumen diameter, branching complexity, tortuosity, caliber heterogeneity, depth distribution, vessel continuity, and perfusion asymmetry. In inflammatory disease, such parameters may provide a readout of subclinical activity or early treatment response; in neoplasia, they may help distinguish indolent from aggressive lesions, stratify biologic behavior, or identify early vascular changes under therapy. The fact that these parameters can be acquired repeatedly over time without tissue destruction is especially attractive in dermatology, where lesions are accessible, follow-up is frequent, and therapeutic monitoring increasingly relies on objective surrogate markers. In this regard, the future of angiogenesis imaging may lie not in a single modality, but in the intelligent integration of complementary information: dermoscopy for rapid surface pattern recognition, RCM for near-cellular horizontal tissue detail, D-OCT for perfusion and vascular depth, and LC-OCT for three-dimensional structural mapping of the vascular microenvironment. Established qualitative patterns include arborizing vessels in BCC, glomerular vessels in Bowen disease, regular dotted/papillary loops in psoriasis, polymorphous vessels in many invasive melanomas, and rainbow/vascular clues in Kaposi sarcoma. Emerging quantitative descriptors include D-OCT vessel density and plexus depth in non-melanoma skin cancer and psoriasis, LC-OCT loop morphology in psoriasis, and three-dimensional segmentation feasibility. Hypothetical biomarkers without adequate evidence of practical benefit include melanoma vascular heterogeneity as a surrogate of Breslow thickness or metastatic risk; vessel density/tortuosity cut-offs as monitors of Bowen disease; routine subtype assignment of BCC by branching metrics; optical separation of hypertrophic actinic keratosis from early squamous cell carcinoma; and OCT staging of Kaposi sarcoma.

At the same time, the present review also underscores the limitations of the field. The literature remains heterogeneous in study design, lesion selection, imaging protocols, and outcome reporting, and most available studies are exploratory, single-center, and based on relatively small numbers of lesions. Vascular terminology is not fully standardized across modalities, and some reported vascular descriptors may reflect shared inflammatory or hemodynamic responses rather than disease-specific angiogenic signatures. Technical limitations also remain relevant. Hyperkeratosis, ulceration, motion artifacts, crusting, or marked pigmentation may reduce image quality and hinder vascular visualization, particularly in deeper or smaller-caliber vessels. RCM is limited by penetration depth and by the difficulty of reconstructing full vascular trajectories over depth; D-OCT, while powerful for perfusion mapping, may not resolve the smallest structural details of angiogenesis; LC-OCT, despite its excellent resolution, still has limited penetration compared with deeper OCT-based systems and requires operator expertise for acquisition and interpretation. Furthermore, as this work was designed as a review, it was intended to map and synthesize the available evidence rather than to generate pooled estimates of diagnostic accuracy or prognostic performance. Accordingly, the current data should be interpreted as a conceptual and translational framework rather than as definitive evidence for routine implementation of angiogenesis-oriented imaging algorithms in all dermatologic settings. Large prospective validation studies are required, with predefined vascular lexicons, reader blinding, histopathologic or outcome reference standards, adjacent-site controls, and reproducibility metrics.

Despite these limitations, the trajectory of the field is clear. Non-invasive vascular imaging is progressively moving dermatology away from a purely surface-based interpretation of vessels and toward a more integrated view of angiogenesis as a measurable, dynamic, and potentially targetable component of disease biology. In this evolving landscape, LC-OCT appears particularly well positioned because it provides a rare combination of near-histologic resolution, vertical and horizontal visualization, volumetric acquisition, and repeatability over time. Its greatest contribution may not be to replace dermoscopy, RCM, or D-OCT, but to serve as a translational bridge between them, linking the immediate recognizability of dermoscopic vascular patterns, the cellular detail of confocal microscopy, and the functional perfusion data of D-OCT into a unified model of cutaneous vascular phenotyping. If future studies succeed in standardizing vascular descriptors, validating quantitative metrics, and correlating imaging findings with histopathology, molecular markers, and treatment outcomes, LC-OCT and related multimodal approaches may become central tools not only for lesion diagnosis, but also for biologic stratification, therapeutic monitoring, and precision vascular dermatology.

Plexus depth varies among diseases in a biologically intelligible way, but published numbers are few and method-dependent. Could it serve as a quantitative parameter of progression or invasiveness? Only as part of a depth-stratified panel, and only after site-adjusted reference values exist. That ambiguity is why Themstrup-type scoring [30] of vessel shape at fixed planes provides morphological information at specified depths, although superiority to a single depth measurement has not been established, and why plexus depth is graded as an emerging, not established biomarker.

Non-specific vascular change is the main threat to any angiogenic biomarker. Inflammation, wound remodeling, ultraviolet injury, and edema all increase vessel visibility. Multimodal reading reduces that threat when architecture is used as a filter: regularity and papillary confinement argue for inflammatory angiogenesis; hierarchical stromal wrapping argues for BCC; chaos plus lacunae plus deeper atypical shapes argue for invasive melanoma or endothelial neoplasia. LC-OCT shows whether vessels respect or disrupt the dermo–epidermal junction and whether they travel inside papillae, around tumor islands, or as a disordered mesh. D-OCT shows whether those structures are perfused and at which standardized depth. Dermoscopy alone cannot make that distinction. The incremental value of combining the three tools is greatest when the clinical question is “what kind of angiogenesis is this?”, not merely “are there more vessels?”. Even so, prospective reader studies that pit multimodal sets against dermoscopy alone, with histopathology as reference and with inflamed mimics in the test set, are still missing. Until those studies exist, we describe an improvement in interpretive specificity, not a measured diagnostic gain.

## 5. Limitations and Strengths of the Study

Several limitations should be acknowledged when interpreting the present review. First, the available literature remains heterogeneous in terms of study design, sample size, lesion type, imaging protocols, and outcome reporting, which limits direct comparison across studies and precludes formal quantitative synthesis. Second, many of the published reports on LC-OCT and D-OCT in dermatology are exploratory, single-center, or case-series based, often focusing on highly selected lesions rather than broader real-world cohorts. Third, vascular descriptors are not yet fully standardized across studies, and terminology may vary depending on the imaging modality used, the disease context, and the authors’ interpretive framework. In addition, some of the vascular features visualized by LC-OCT may not be entirely disease-specific, but may instead represent shared responses to inflammation, tissue remodeling, or local hemodynamic adaptation. Technical factors must also be considered: hyperkeratosis, ulceration, motion artifacts, and marked pigmentation may reduce image quality and hinder the visualization of deeper or smaller-caliber vessels. Finally, because this work was conceived as a review, it was designed to map the existing evidence rather than to generate pooled estimates of diagnostic performance or prognostic accuracy. Accordingly, the findings should be interpreted as a conceptual framework for angiogenic phenotyping rather than as definitive evidence for routine clinical implementation. The protocol was not registered; formal tool-by-tool risk-of-bias scoring of every primary study was not performed; illustrative figures come from author archives and are not a statistical sample; some stage- or subtype-specific statements are cautious inferences from consistent descriptions rather than findings of dedicated comparative trials; English-language restriction may have omitted relevant work; shared hemodynamic responses to inflammation can mimic disease-specific signatures; technical failure rates in hyperkeratotic, ulcerated, pigmented, or moving lesions remain under-quantified. Strengths of the present review include that it addresses cutaneous angiogenesis with an explicitly multimodal framework; it distinguishes primary from secondary angiogenic mechanisms and organized from chaotic architectures; methods describe databases, search concepts, and the scope of the narrative synthesis; disease selection is justified on biological grounds; illustrative images require confirmation of provenance and panel assignments; biomarker claims are graded as established, emerging, or hypothetical; and the discussion identifies uncertainties in stage and disease comparisons, plexus depth, and the specificity of vascular change.

## 6. Future Directions

Advances in LC-OCT technology and analysis have the potential to transform angiogenesis-focused dermatology. Integration with artificial intelligence and machine learning algorithms could enable automated segmentation and quantitative morphometrics, improving reproducibility and facilitating early detection of angiogenic shifts. Multimodal approaches combining LC-OCT with techniques such as dynamic OCT, photoacoustic imaging, hyperspectral OCT, or OCT angiography may provide complementary functional and oxygenation data, yielding comprehensive vascular phenotyping. Vessel density and tortuosity in Bowen disease, OCT staging of Kaposi sarcoma, and aggressiveness markers in melanoma should remain labeled as research hypotheses.

Longitudinal studies are warranted to validate LC-OCT-derived metrics as predictive or prognostic biomarkers, particularly in high-risk populations or in monitoring response to targeted therapies. Potential research applications include studying vascular changes in UV-exposed skin. Imaging findings currently do not establish an indication for preventive topical anti-VEGF treatment. Additionally, integration into clinical workflows requires optimization of acquisition protocols to mitigate motion artifacts and improve imaging in hyperpigmented or hyperkeratotic lesions. Plexus depth should be reported with adjacent skin controls and standardized en face planes (150/300/500 μm). Future reader studies should include inflamed mimics so that disease-specific patterns can be tested against non-specific remodeling rather than only against healthy skin.

## 7. Conclusions

Dermoscopy, D-OCT, and LC-OCT provide complementary information about cutaneous vascular morphology. LC-OCT contributes three-dimensional structural detail, whereas D-OCT provides motion-contrast visualization of perfused vessels; structural LC-OCT alone does not measure perfusion dynamics. The reviewed studies support exploratory vascular characterization across selected skin diseases, but evidence is insufficient for routine biomarker-guided staging, prognostication, or treatment selection. Standardized descriptors, reproducibility studies, and prospective validation against histopathology and clinical outcomes are required.

## Figures and Tables

**Table 1:** Representative LC-OCT and OCT-angiography vascular features in dermatology. Evidence grade added conceptually in the revised text (not a formal GRADE assessment): qualitative established patterns versus emerging quantitative metrics versus hypothetical biomarkers. Plexus depth is emerging, not established.

Condition	Typical Vascular or Structural Features on LC-OCT/OCT-A	Clinical Relevance
Melanoma	Irregular dermoscopic vessels; D-OCT vascular morphology; LC-OCT structural vessel–tissue relationships	Correlates with Breslow thickness in limited series; not validated as a staging tool
Basal cell carcinoma	Arborizing dermoscopic vessels; D-OCT vascular observations; LC-OCT tumor lobules and adjacent vascular spaces	Adjunctive assessment; vascular metrics alone do not establish subtype or margins
Actinic keratosis/SCC	Dilated glomerular vessels; interrupted DEJ in SCC; bizarre nodular clusters	Supports structural assessment; vascular treatment-response prediction remains exploratory
UV-damaged skin	Rapid rarefaction followed by serpiginous recovery; subclinical tortuosity	Experimental observations; preventive thresholds for chronic photodamage are unvalidated
Psoriasis	Elongated papillae with dilated dark capillaries; regression post-biologics	Activity scoring; biologic response tracking

## Data Availability

No new data were created or analyzed in this study. Data sharing is not applicable to this article.

## Abbreviations

The following abbreviations are used in this manuscript:

LC-OCT	line-field confocal optical coherence tomography
D-OCT	dynamic optical coherence tomography
